# P-1654. Factors affecting vancomycin and piperacillin/tazobactam-induced nephrotoxicity

**DOI:** 10.1093/ofid/ofae631.1820

**Published:** 2025-01-29

**Authors:** Ruhul Munshi, Mollie VanNatta, Muhammad Haris Khan, Mohammad Alam, Alexandre E Malek

**Affiliations:** LSU Health Shreveport, Shreveport, Louisiana; Ochsner LSU Health Shreveport, Shreveport, Louisiana; LSU Health Shreveport, Shreveport, Louisiana; Louisiana State University Health Sciences Center, Shreveport, LA, USA, shreveport, Louisiana; LSU Health Shreveport, Shreveport, Louisiana

## Abstract

**Background:**

The concomitant use of vancomycin (Vanc) and piperacillin-tazobactam (Pip/Tazo) is one of the most common combinations utilized in infectious disease field. But, studies have hinted at an increased risk of nephrotoxicity. However, the specific factors leading to the development of acute kidney injury (AKI) remain uncertain. In this study, we sought to elucidate the potential factors contributing to AKI in pts who received both Vanc and Pip/Tazo

**Figure:**
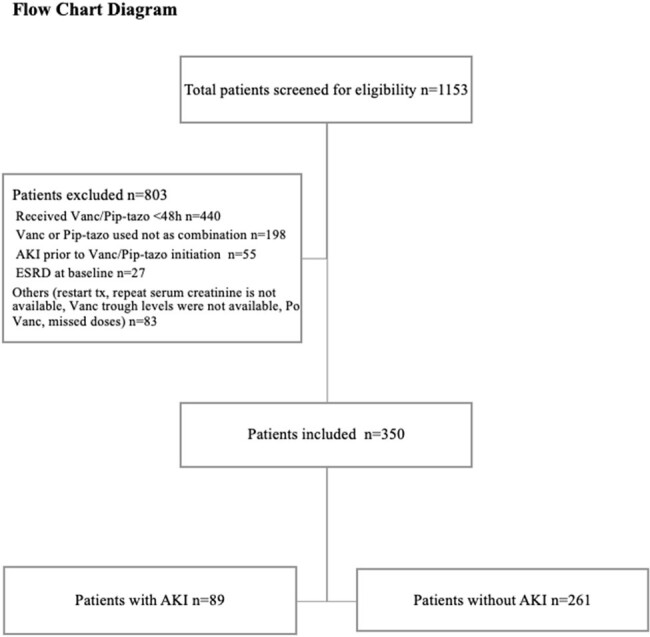

**Methods:**

We performed a retrospective study of adult pts admitted at the Ochsner LSU Health Shreveport - Academic Medical Center between May 2019 and March 2023 who received at least 48h of Vanc and Pip/Tazo combination. Exclusion criteria and number of eligible pts are outlined in the flow chart. Demographics, calculation of Vanc levels and AUCs, and information on the treated infectious syndromes were all collected. AKI was defined according to RIFLE and AKIN criteria. We then compared pts who developed AKI versus those who did not.

**Figure:**
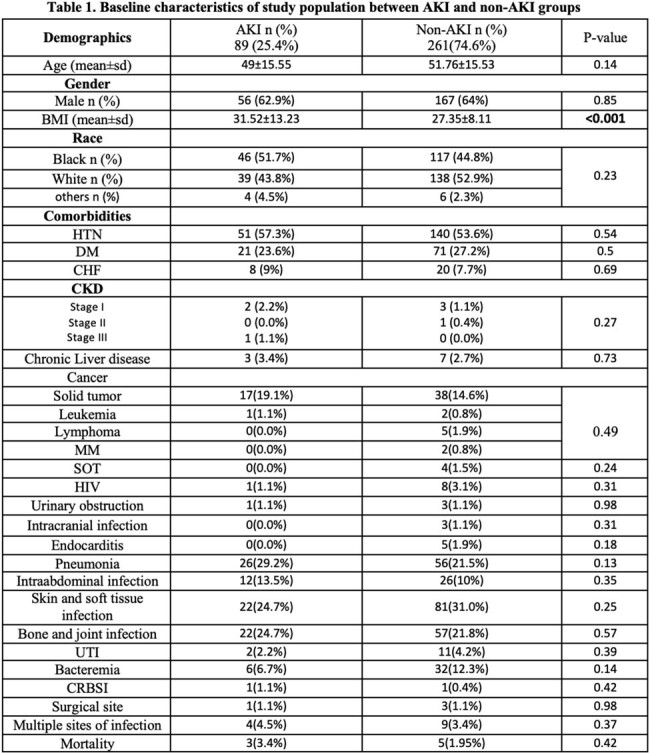

**Results:**

Of 1153 pts, we included 350 pts who met the inclusion criteria. Pts were divided into AKI group (n=89, 25.4%) and non-AKI group (n=261, 74.6%). Pts’ demographics at baseline were comparable between the two groups, except obesity was more prevalent in pts in the AKI vs non-AKI groups, with mean BMI of 31.52 vs 27.35, respectively, p=value < 0.001 (Table 1). There were no statistically significant differences between the two groups in terms of concomitant use of potentially nephrotoxic agents, except the use of IV contrast was significantly higher in the AKI group (31.5%) compared to the non-AKI group (18.0%) with p=0.008 (Table 2). On the univariate analysis, the mean duration of combination therapy was higher in the AKI group vs non-AKI group, 4.65 vs 4.04, respectively, p=0.01. Vanc loading and maintenance doses, AUCs, and trough levels were significantly higher in the AKI group (Table 3). However, logistic regression analysis showed that IV contrast was an independent risk factor for AKI (OR=2.4, 95% CI=1.14-5.03, p=0.02).

**Figure:**
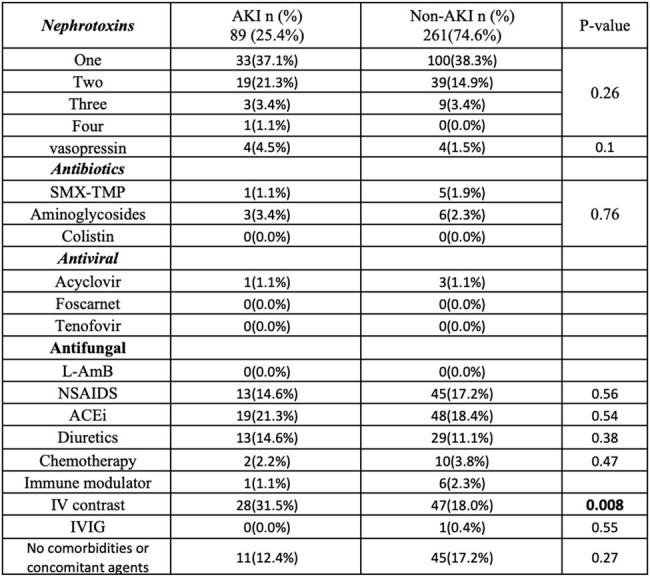

**Conclusion:**

Our data demonstrated that IV contrast is significantly associated with an increased risk of nephrotoxicity in pts receiving Vanc and Pip/Tazo combination therapy. The data support that the combination of vanc and Pip/Tazo is not as nephrotoxic as initially thought.

**Figure:**
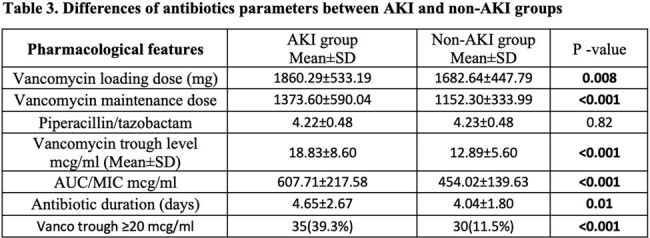

**Disclosures:**

**All Authors**: No reported disclosures

